# At the End of Every Pandemic: Beginning a Pandemic Playbook to Respond to the Next

**DOI:** 10.3389/fpubh.2022.838561

**Published:** 2022-04-27

**Authors:** Peter G. Goldschmidt

**Affiliations:** World Development Group, Inc, Bethesda, MD, United States

**Keywords:** COVID-19, management, pandemic, playbook, planning, preparedness, response, system

## Abstract

The world was unprepared for COVID-19. Pandemics can unfold quickly; faster than governments can respond, unless they have maintained a realistic pandemic playbook. As the world ahead becomes ever-more complex, such playbook becomes ever-more necessary. This article not only describes the importance of a pandemic playbook but also a system to maintain it. A pandemic playbook both (1) specifies what is needed to respond to a pandemic and (2) provides a lens through which to identify measures that will keep people safe and society secure. The plays in the book are thought-though policies and strategies and corresponding implementation plans. The process of developing a playbook is as important as the product. Any playbook must be fit for purpose in the context of the times in which it is to be used. Above all, it must contain realistic policies and plans that can actually be implemented and can realize their intended effects. Achieving this goal requires (1) repeatedly exercising the playbook so that people know what to do when they need to do it and (2) evaluating results and updating the playbook to keep it relevant and current. Necessarily, to bring ideas alive, this article illustrates them with reference to COVID-19 and earlier pandemics, but it is not intended as a playbook for responding to the next pandemic; nor a postmortem on responses to COVID-19. Instead, it describes actions to take now to be ready when the next global pandemic strikes, so that policy decision-makers will not be lamenting “we should have done that.”

## Introduction

### Unheeded Warnings

Pandemics end; the threat of pandemics never does. Epidemics have occurred since the dawn of human history when people started to live in close proximity to one another, and with animals. In more recent centuries, increasing societal complexity and globalization have conspired to promote global pandemics. Their origin remains uncertain; their ending, obscure. For decades, experts (1) have warned that the next global pandemic was only a matter of time (1, 2) and (2) have identified needed preparedness upgrades (3–5). Despite multiple pandemics between 1918 and 2019, these warnings went mostly unheeded. Historians have provided insights into past pandemics (6); epidemiologists have described lessons learned to fight the next one (7). Pandemic management-games have revealed weaknesses in and corresponding improvements to preparedness (8). Unfortunately, policy decision-makers have largely ignored these recommendations. Developing a realistic playbook requires leadership; when a pandemic strikes no playbook can overcome lack of leadership. When COVID-19 (a pandemic caused by a novel coronavirus) struck preparedness consisted mostly of inadequate, out-of-date reports and plans on shelves that focused on a century-old enemy, pandemic influenza; where plans existed, leaders mostly ignored them. A few months before the covid pandemic began, the USA was reported to be the nation that was the most prepared to respond to a pandemic (9). Events soon proved otherwise.

### Government Failings

Most governments were unprepared to respond to covid, lost valuable time in mobilizing a realistic response, and unleashed a potentially avoidable social catastrophe (10). Leaders in many countries initially underestimated the pandemic's severity; in others, simply resisted introducing extraordinary measures that might frighten people or cause economic disruption (11). They not only failed to base policy on sound science but also acted contrary to what was needed, which further reduced already low levels of trust in institutions, even as the accompanying infodemic further eroded trust in public health authorities (12).

### New Beginnings

This article begins as a pandemic ends (exemplified by covid), to create a vision of a comprehensive, coherent, digital-age pandemic playbook (PPB) designed to counter the threat of the next global pandemic. As societies become more complex and the world continues to become more interconnected, the next global pandemic could pose an existential threat. Pandemic management requires an entirely new, digital-age approach. The PPB system described here represents a plan to plan, a playbook for developing a playbook; not a detailed blueprint specific to any country.

## Pandemic Playbook

### Need for Playbook

A pandemic playbook (PPB) both (1) specifies what is needed to respond to a pandemic and (2) provides a lens through which to identify measures that will keep people safe and society secure. Modern life has increased the threat of pandemics from: (1) animal viruses jumping to humans (e.g., covid), (2) variants of endemic diseases (e.g., 1918 pandemic), and (3) industrial accidents, and bioterrorism or bio-warfare. Globalization, international trade, air-travel and tourism, and labor migration and other recent changes and trend-accelerations have led to the spread of novel zoonotic diseases and known (endemic) diseases (13). Pandemics unfold quickly; often faster than appropriate responses can be formulated. The plays in the book are thought-though policies and strategies (PASS), and corresponding implementation plans to respond to various plausible scenarios within a given contextual background. A realistic PPB allows (1) difficult policy choices to be considered in advance (and subjected to public scrutiny) and (2) quick, appropriate, and decisive response when a pandemic threatens or strikes. A PPB supports policy decision-making; it does not diminish the need for leadership or constrain policy choices.

### Past Pandemic Playbooks

The concept of a PPB springs from practical experience in managing plague (14). Administrative organizations were first created to manage the sanitary system during plague outbreaks in the fourteenth and fifteenth centuries; most successfully in north-central Italian city states. Now-familiar non-pharmaceutical interventions (NPI) emerged as Renaissance pubic health policies (14, 15). Preventive measures to contain a plague outbreak (in Sardinia in 1582–83) were introduced by the Neapolitan physician Quinto Tiberio Angelerio (who had practiced in Sicily during the 1575–76 epidemic), including disinfection of houses in which people had died of the disease and the use of dry heat to sterilize objects (14). Angelerio's 57 instructions, subsequently published in 1588 (16), became the basis for subsequent pandemic response management (PRM) (14). Many of them would become familiar during covid: lock-downs, allowing only one person per household to go shopping, forbidding meetings and entertainments, and maintaining social distancing (aided by mandatory use of a white cane of about 2 m) (14, 15).

### Country-Specific Pandemic Playbook

A PPB is necessarily country-specific: (1) no country can rely on others or any international organization to do right things right at the right time and (2) plays in the book need to be consistent with a country's prevailing culture, socio-political traditions, and circumstances. Regional governments may want PASS tailored to local circumstances while a central government may be concerned with uniformity of national response within a global context. A realistic national PPB requires parallel PPB appropriately duplicated downstream maintained by regional and local governments and other organizations, particularly those that are part of critical infrastructure such as hospitals. Each PPB contains well-thought through responses to plausible what-if scenarios that might arise from a pandemic, including the simultaneous occurrence of geo- or weather-related natural disasters and/or other public health emergencies. Effective PRM requires not only policy responses focused on health care services but also those in pertinent socio-economic sectors in order to make health-related policies work (17). For example, stay-at-home orders require consideration of income maintenance, food delivery, evacuation of sick people, and so on; all of which, in turn, require corresponding policies and strategies, implementation plans, and the existence of infrastructures needed to make them work. Nevertheless, PPB (1) should plan only for what needs to be planned for and (2) should delineate broad policies rather than detailed procedures. Not all situations require policy responses to be thought-out in advance for at least 2 reasons: (1) the situation on the ground can change too quickly for meaningful advance planning or (2) appropriate policies can be formulated when needed, their potential consequences can be evaluated quickly, and appropriate implementation plans can be devised in near-real time. In such cases, only scenarios, questions, and pertinent knowledge need to be identified in advance.

### Creating and Maintaining Realistic Playbook

A continuous, iterative planning process is at least as important as the resultant plan because its enduring mechanism can create a common understanding among stakeholders and facilitates downstream communications. Beginning at the end of a pandemic has the advantage of lessons learned, including real-world insights into (1) effectiveness of policies and messaging, (2) adequacy of planning, plans, and infrastructures, (3) data needed for informed decision-making, and (4) accuracy of models. Interactive contingency planning can help to develop realistic plans; especially by counteracting potential optimism bias. Innovative solutions can include both (1) mathematical models (a broad term encompassing traditional and AI analytics, neural-networks, Baysian networks, crowd-sourcing, prediction-markets, computer simulations, etc) and (2) interactive, role-playing techniques (management gaming, policy Delphi, etc). The days of a few experts sitting around a conference room table to produce a document that sits on a shelf—Renaissance-era technology—should be long gone. A digital-age PPB is a user-friendly computer-networked decision support tool. The digital-age greatly enhances the ability to maintain a realistic PPB and to implement its PASS. In the future, AI analytics, near-real-time dashboards, and other digital-age technologies can be expected to play an ever-expanding role in gauging and guiding policies.

### Selecting Scenarios and Assessing Alternatives

Planning steps include (1) deciding what scenarios should be included in the PPB, (2) describing, for each, relevant aspects of the current contextual background and the state of pertinent infrastructures, (3) assessing alterative PASS for responding to chosen scenarios, and (4) planning for effective implementation of preferred PASS as a basis for future action (using processes similar to those for selecting and prioritizing PASS). Realistic PASS (1) accommodate infrastructure limitations (spurning aspirations of what might exist), (2) meet people where they are (not where planners would like them to be), and (3) are most effective when they focus on harm reduction by advising people what they should do (e.g., to remain safe, rather than what they should not do). Alternative PASS are regularly (1) assessed in relevant detail, including expected second and subsequent order effects on all pertinent socio-economic sectors and (2) analyzed iteratively to identify potential negative effects and to develop mitigation policies that themselves need to be similarly analyzed. While such work may seem Herculean, especially when undertaken to manage potential future scenarios, it is nevertheless practical and can have enormous payoff in terms of preparedness and eventual outcomes. Decisions inevitably involve value-laden choices, often among unenviable options: choosing the lesser of evils. This reality creates an ongoing tension among what is best, what is practical, and what is right. While expertise is required to assemble, analyze, and assess scenarios, PASS, and implementation plans, choices among alternatives and trade-offs require stakeholder input and decisions by political leaders who are accountable for them. Lack of accountability has corrosive effects on society, including (1) allowing leaders to act in their personal interest rather than choosing to do the right thing, (2) reducing morale and decreasing trust in government, and (3) in the long-term, threatening social stability.

## Pandemic Management

**Figure 1:**
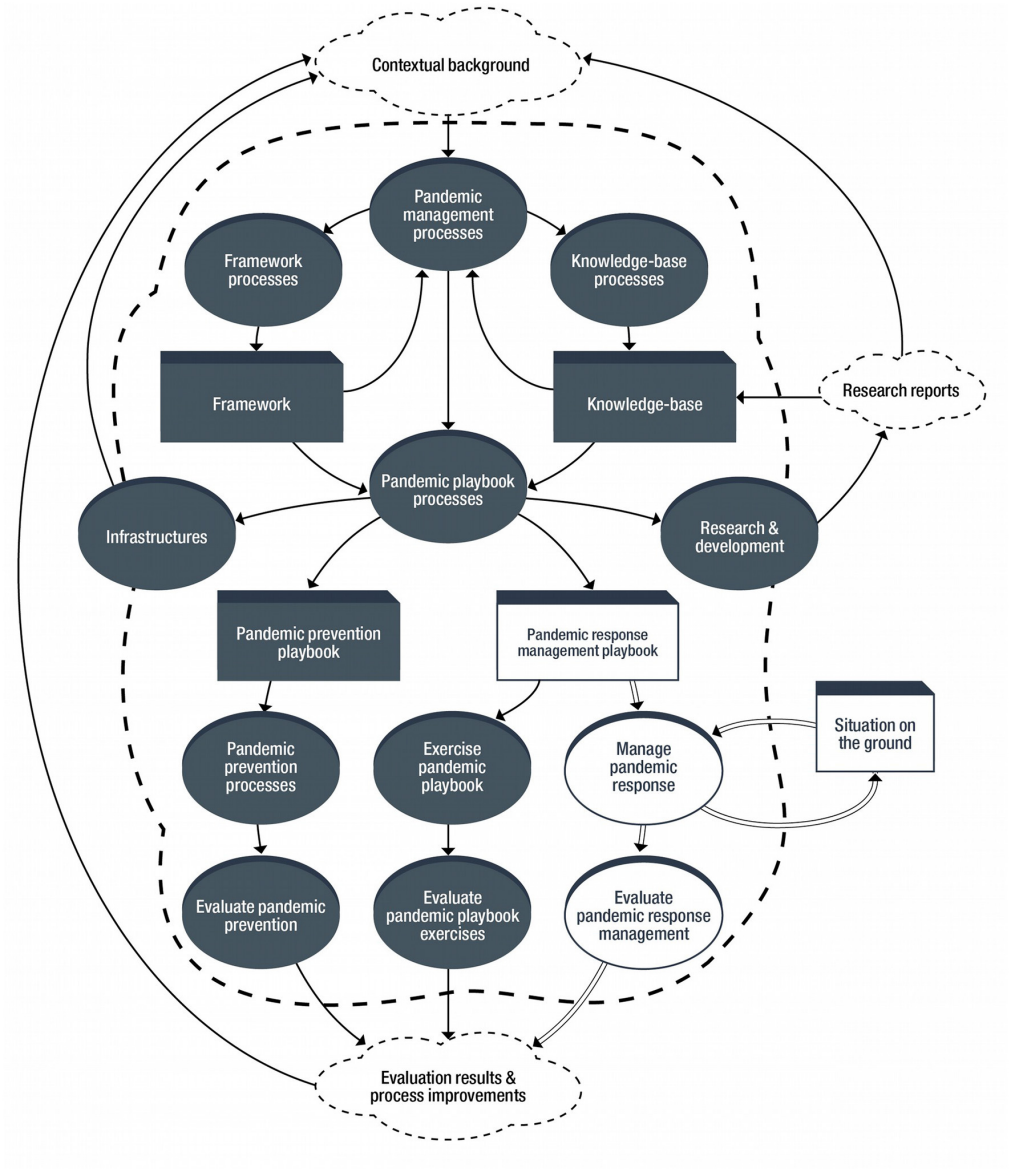
Pandemic playbook system.

The overarching goal of pandemic management is to end pandemics before they start or, failing that, to prevent social catastrophe when they occur. Achieving this goal requires a separate, permanent, appropriately-resourced national Pandemic Response Management Agency (PRMA) that manages the system that maintains and implements the PPB. See Figure 1. Such PPB system (PPB-S) may be operationalized as an aspect of broader resilience management or whole-society security (18). The PRMA (1) is independent, (2) coordinates the work of, other government agencies and organizations that play a role in achieving its mission, and (3) reports directly to the country's President (or highest level government official). The national public health authority is not responsible for the PPB-S because pandemic prevention and response (1) is a national security issue and (2) involves all socio-economic sectors. Interlocking teams manage PPB-S subsystems: (1) central management, (2) pandemic framework (which organizes the goals, scope, and contents of the PPB), (3) knowledge-base, (4) pandemic prevention and (5) pandemic response management. They embody a wide-variety of relevant stakeholders and encompass all pertinent perspectives; some members may serve on multiple teams. A standing executive board—Pandemic-Central—manages pandemic response.

## Pandemic Framework

### Framework Purposes and Principles

The pandemic framework: (1) organizes interrelated concepts into a functional whole, (2) provides a data-driven relevant picture of reality, (3) creates structures for interactive, long-term planning, (4) describes fundamental undergirding objectives and policies, (5) forms the foundation for PPB-S design and management, and (6) specifies models to facilitate decision-making. The framework is constructed around 2 main principles: (1) heath in everything and (2) one health. Health in everything means that (1) every socio-economic sector influences population health and (2) policy-analyses should always consider population health impacts of every socio-economic sector (19). One health (1) represents a collaborative, multi-sectorial, and trans-disciplinary approach and (2) recognizes that the health of humans is intimately connected to the health of animals and their shared environment (20). The framework is used (1) to specify what needs to exist for effective PRM, (2) to compare what should exist to what is believed to exist and what actually exists, and (3) to prioritize closing gaps, bringing perceptions in line with reality and reality in-line with specified requirements.

### Framework Development and Maintenance

Creating a pandemic framework requires (1) establishing a framework for the framework and (2) building on what exists. Maintaining frameworks is an iterative process that requires the engagement of stakeholders and attention to their various viewpoints. Frameworks are (1) represented in machine objects and artifacts and (2) depicted in human-readable, user-friendly formats (e.g., info-graphics and flowcharts), and traditional narratives (e.g., standard operating procedures). Frameworks are revised based on (1) refined thinking, research, and experience, (2) changes in and expected evolution of the contextual background, (3) feedback from quality management systems, and (4) results of exercises and evaluations.

## Pandemic Knowledge-Base

### Purpose, Organization and Management

The knowledge-base represents a standard-view of what is known that is relevant to pandemic management. It provides access to scientifically-sound knowledge and can generate summaries on-demand. The pandemic framework (1) determines the scope of the knowledge-base, (2) dictates its contents, and (3) shapes knowledge-base specifications (which are revised whenever the framework is revised and contents mapped accordingly). Maintenance of framework topics could be distributed internationally, but well-resourced countries may want to rely instead on distributing them among domestic institutions. Given access to knowledge that applies worldwide, less-well-resourced countries can focus on country-specific knowledge.

### Sources of Knowledge

Sources of knowledge are as varied as necessary to meet PPB-S objectives. They may include (1) published research reports, (2) compilations (e.g., international standards, regulations, and good practices), (3) projects to fill knowledge gaps, (4) PPB-S analyses and evaluations, (5) results of mathematical models, (6) structured inquiries of experts (e.g., to estimate parameters when research studies are not practical), (7) health information factories (21), (8) surveillance systems, and (9) social media postings. When a pandemic strikes processes are geared-up to keep pace with the accompanying scidemic: multifold increases in the number of published scientific papers and/or the rate of their publication compared to the background rate. The covid scidemic was fueled by lavish government funding; it overwhelmed scientists. Moreover, AI-based tools were of limited value in managing it (22). In about the first 15 months, over 250,000 full-text articles were logged into a covid citation-base (23). Initial evidence suggests that much covid research was a waste of money (24, 25) and publications untrustworthy or of poorer quality than usual (25–29); usual quality is itself poor (30).

### Maintaining Knowledge-Base

Knowledge-base processes operate continuously, in near-real time:

(1) making accessible know-bits (kernels of knowledge), (2) storing meta-data about each know-bit (including type and source, its quality or scientific-soundness, and how it is classified according to the framework), and (3) prioritizing knowledge-gaps (based on what needs to be and what is known with required scientific-soundness). Processes are enabled by digital-age technologies, including machine learning and other advanced analytics, which can be expected to feature ever-more prominently. Managing research includes (1) funding projects to fill knowledge gaps, (2) maintaining an inventory of research projects to be launched when a pandemic strikes, and (3) supporting mechanisms to launch such projects quickly and to unleash latent data collection systems. Managing information requires (1) operating factories to manufacture information products that are essential for pandemic management decision-making, (2) maintaining curated data sets, and (3) arranging for reliable supplies of fit-for-purpose data. Users' feedback on knowledge-base utility is instrumental to its improvement.

## Preventing Pandemics

Preventing pandemics requires worldwide collaboration (1) to prevent the emergence of novel pandemogens (e.g., by supporting international conservation programs, managing the world's ecosystems, and maintaining bio-diversity) and (2) to recognize the emergence of a pandemogen. Once a novel pandemogen starts to circulate among a human population, a pandemic is almost certain to follow. The only defense is surveillance to identify the pandemogen sufficiently early and pre-established mechanisms to stop it from spreading (and, potentially, extinguishing it). Once a pandemogen has spread beyond its source, countries must gear up their response capabilities.

## Pandemic Response Management

### Pandemic Preparedness

**Figure 2:**
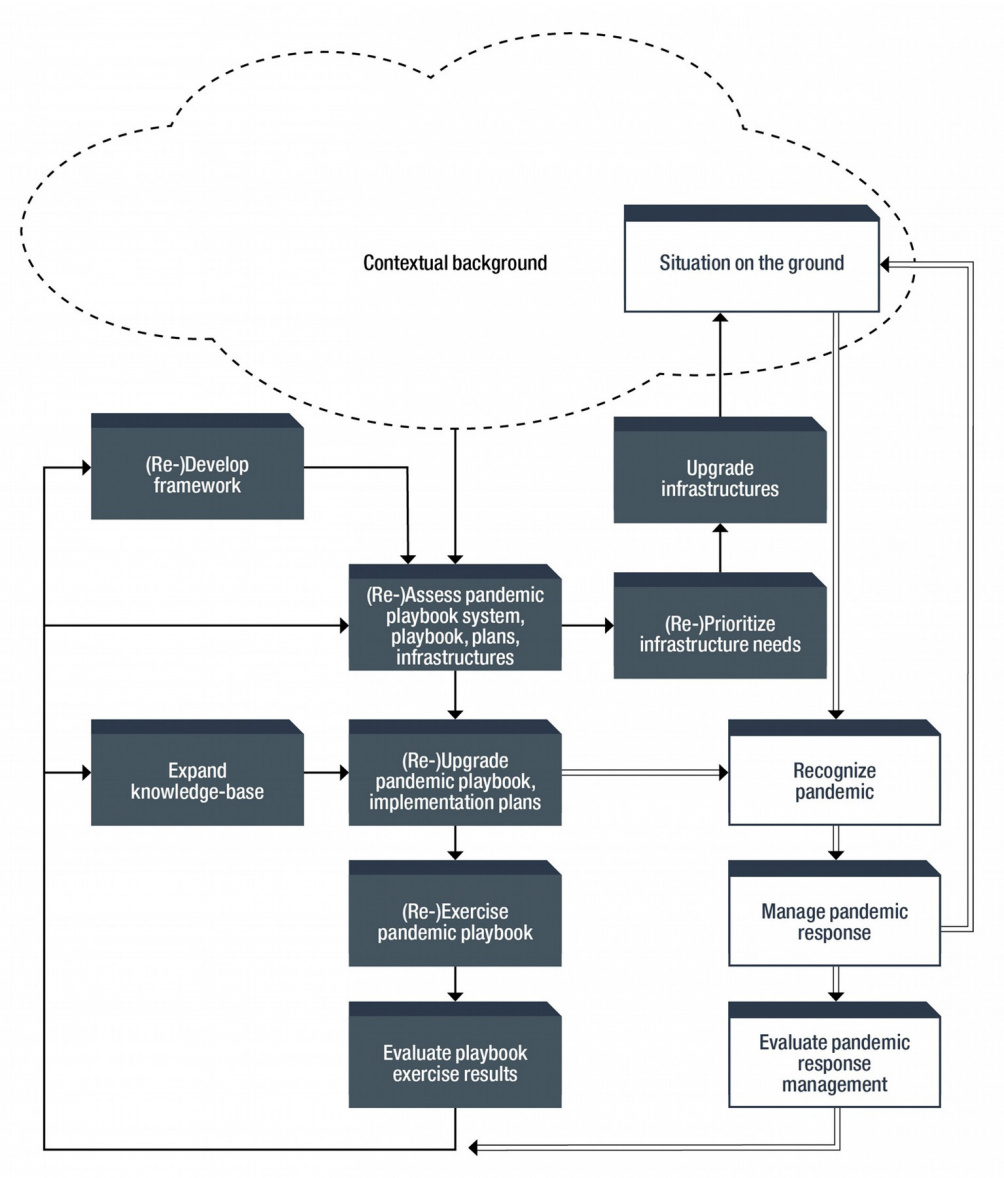
Pandemic response management flowchart.

Pandemic response management (PRM) is an active process. It involves (1) *preparedness* (to be ready to respond rapidly and without which it is already too late to respond effectively), (2) *surveillance* (to know when to respond) (3) *action* (to respond decisively and effectively while minimizing socio-economic impacts of controlling the pandemic), and (4) *evaluation* to strengthen future pandemic management (based on results of exercises and lessons learned from pandemics). See Figure 2. Achieving goals requires not only (1) maintaining a realistic pandemic playbook but also (2) ensuring the contextual background promotes preparedness and response. No amount of preparedness or planning can overcome a lack of or failed leadership and/or inappropriate or inconsistent pubic health messaging. Political authorities should defer to experts and should prefer science-based policies. As covid demonstrated, such approach cannot be taken for granted.

### Public Health Messaging

Public health messaging is essential (1) to educate people about pandemic preparedness and (2) when a pandemic occurs, to communicate the current situation and corresponding policies, restrictions, and precautions. Public health messaging is best when (1) constant, consistent, coherent, and comprehensible), (2) authoritative (based on science), (3) delivered by a qualified professional, (4) backed by politicians leading by example, (5) out in front of public opinion (rather than following behind or out-of-touch), and (6) realistic (meeting people where they are). In normal times, messaging involves informing the public of the ever-present threat of a pandemic, how authorities are prepared to respond, and what to expect when a pandemic occurs. During a pandemic, saving lives depends on (1) communicating what are the right things to do and reasons for doing them, (2) encouraging people to adopt sensible precautions (think hand washing, masking, and social distancing), and (3) managing the accompanying infodemic [which is characterized by a rapid spread of an overabundance of information, misinformation, disinformation, and fake-news, can spread faster than a pandemogen, and complicates public health messaging (31)].

### Scanning Contextual Background

Effective pandemic management requires an up-to-date picture of the contextual background: the setting (time, place, and environment) in which events occur; specifically, the nature and characteristics of society in a particular place during a particular period. The contextual background encompasses, but is different from, the situation on the ground (SOG): a momentary aspect of the contextual background: circumstances that exist currently in a particular place and what is actually happening or unfolding there; captured in (near) real-time. Pertinent characteristics which best reflect a particular context depend on the purpose for understanding how it shapes and/or is shaped by prevailing conditions, events and effects, actions and results, and trends and forecasts. A PPB is shaped by and shapes the contextual background. Constantly scanning the contextual background is essential to ensure that appropriate infrastructures are available with required capacity (as they cannot be readily upgraded when a pandemic strikes). Essential infrastructures include (1) public health programs, (2) health care services, (3) supply chains and stockpiles (for drugs, devices, personal protective equipment, etc), and (4) research and other functional ecosystems. Changes to infrastructures (including those resulting from closing preparedness gaps and remedying revealed social disparities) require revising the PPB to keep it current and relevant.

## Pandemic Response

### Monitoring Signals for Pandemics

Preparedness means (1) being constantly alert to the possibility of a pandemic and (2) knowing when to activate PRM and the appropriate level of activation. Timing is the key to success. By waiting to see if a pandemic is really underway, it may already be too late to suppress the pandemogen. Responding to a pandemic that does not exist also has consequences but they pale in comparison to those of the alternative. Preventing the spread of a pandemogen is not only the first line of defense, it is the only defense (32). Alertness encompasses (1) direct signals (e.g., from public health surveillance systems), (3) proxy-signals (from a wide-variety of sources, such as social media postings), and (3) warnings from international organizations. A country might adopt a traffic-signal alert and response scheme; the color indicating the alertness level and corresponding actions for (1) implementing pertinent PPB PASS and (2) messaging the pubic about pandemic restrictions and precautions.

### Containing Pandemogen Spread

When a pandemic threatens or strikes Pandemic-Central swings into action. When this time comes, there is no guarantee that PPB PASS would exactly match the unfolding pandemic picture. Containing spread as quickly and as completely as possible is the overarching goal. Minimizing the number of cases is important: (1) to prevent health services from being overwhelmed by a surge in pandemic patients, especially those who need life-saving care, and buying time to develop pharmaceuticals, (2) to minimize the number of deaths and the possibility of long-term health effects among survivors, (3) to reduce the risk of the emergence of more dangerous variants of the pandemogen (which could undermine containment efforts, could compromise immunity from past exposure to the pandemogen or vaccination, and could prolong the pandemic worldwide), and (4) to decrease economic impacts, social disruptions, and other knock-on effects. Throughout the pandemic, Pandemic-Central adjusts plays in the book to match the SOG. Success or failure is predominantly judged by the number of excess deaths that occurs in a population (in a given period); first used with plague, more than 350 years ago (33). Active management of a global pandemic, from beginning to end, was first experienced with covid.

### Phases of Pandemic Response

From a public health perspective, PRM can be divided into 3 phases: (1) beginning, (2) middle-slog, and (3) endgame. Suppression of the pandemogen is the beginning goal, achieved by implementing such NPI as: (1) controlling access (e.g., international borders), (2) limiting movement and mixing (e.g., closures, lock-downs, and socio-physical distancing), (3) testing and case tracing (to isolate infected individuals, to quarantine contacts, and to mop-up outbreaks), (4) mandating or encouraging personal behaviors (e.g., hand-washing and mask-wearing), and (5) modifying and maintaining the built environmental (e.g., ventilation systems, partitions, and surfaces). Countries that fail to respond quickly and effectively find themselves in the middle-slog where mitigation is the goal: slowing the increase in the number of new cases (referred to flattening or bending the curve). The danger in the middle-slog is losing control; then attention shifts to focusing on doing whatever is still possible to slow pandemogen spread and to cope with its consequences. Authorities need to get ahead of pandemogen spread; not to follow hopelessly behind instituting demoralizing stricter policies than would otherwise be necessary. They may race desperately to catch up with many activities amounting to pandemic theater: responses that are only for show and cannot realistically be expected to be effective or are a waste of resources.

### Endgame Management

A global pandemic may end in some countries but continue in others; complicating the endgame. Without effective vaccines or specific treatments managing the endgame poses particularly difficult policy challenges. Endgame policy-shaping dimensions include (1) whether or not a country has suppressed pandemogen spread and (2) whether or not effective pharmaceuticals (principally vaccines) are available. With suppression and vaccines, pandemics can likely be actively managed to a quick end. Without vaccines, countries that fail to suppress spread could be devastated while those that succeed become hermit kingdoms. The availability of a vaccine (1) may encourage the belief that the end is in sight, (2) may facilitate endgame management, and (3) may alter people's behavior, which, in turn, may determine when and how a pandemic ends.

### Mass Vaccinations

While vaccines can slow and, ultimately, can halt the spread of a pandemogen, they may not necessarily hasten the end of a pandemic. With covid, building on years of investment in bio-medicine, Operation Warp Speed in the USA (and a similar initiative in the UK) resulted in the availability of an effective vaccine in less than a year, an unprecedented success. Unfortunately, parallel efforts were lacking to administer the vaccine to everyone in the shortest possible time. Challenges of mass vaccination campaigns include (1) producing enough vaccines (including both manufacturing and supply chain issues), (2) logistics (getting vaccines into people), (3) ethics (who should be vaccinated first), (4) vaccine hesitancy (people not wanting to be vaccinated), (5) vaccine wall (insufficient proportion of the population vaccinated to achieve community immunity), (6) explicit and/or stealth vaccine mandates (e.g., as a condition of employment), (7) vaccine/health passports (e.g., to access services), including fake certificates and test results, (8) breakthrough infections among vaccinated people (and tracking the variants involved), vaccine escape, and determining the need for booster jabs, and (9) sharing vaccines (and/or the technology to produce them) internationally. Variants are wild cards affecting the path to the end. The need to vaccinate everyone as quickly as possible changes the challenge from one of scale to one of kind. With covid, plans to vaccinate everyone on the planet fell short. Worldwide community immunity became a unicorn galloping into the distance.

### Winding-Down and Opening-Up

Challenges of the endgame include (1) planning to relax or remove restrictions in force when the endgame begins, to open-up closed businesses, to allow social events, and so on, to transition to a new normal, (2) deciding vaccination policies, (3) combating pandemic fatigue and maintaining pandemic precautions during the transition period, (4) continuing testing and tracing to deal with outbreaks, (5) caring for pandemogen survivors experiencing long-term effects, and (6) dealing with continuing socio-economic impacts. The transition to the post-pandemic new normal is likely to be gradual, with post-pandemic activities to deal with enduring issues blending into the emerging contextual background. Under a new normal, there are no longer any special efforts to end the pandemic; the pandemogen is now part of the landscape.

## Discussion

### Consequences and Costs

Global pandemics are deadly, costly, and disruptive; they are history shaping events. Their second and subsequent order consequences ripple through the future and far exceed their immediate impacts. Covid has shaken long-held assumption about resilience and adaption and created new uncertainties about the economy, government, geopolitics, and technology. It took millions of lives, and cost tens of trillions of dollars in direct and indirect costs (34), and will likely affect people's lives for generations. Its eventual societal impact may exceed that of the 1918 pandemic. The costs and consequences of the next global pandemic could be even greater. They are more than sufficient justification for allocating substantial resources to pandemic management. How a pandemic ends depends on interrelated, ever-changing factors such as: (1) nature of the pandemogen, (2) population characteristics, and (3) physical and social environments. They create a kaleidoscope of images, which individually or when combined, result in the perception that a pandemic has ended. Pandemics never end because they are an ever-present threat.

### Global Coordination

Ending pandemics requires a coordinated global response: no country is safe until all countries are safe. Covid revealed the failings and limitations of international organizations. A pandemic treaty and contemplated reforms may strengthen their capabilities but are unlikely to change realities of PRM. International organizations can only operate within their charters and policies dictated and resources provided by their member states. They lack enforcement powers and depend on countries voluntary cooperation. At best, they (1) can facilitate prevention of, and alert countries to, the emergence of pandemogens, (2) can provide technical assistance, and (3) can have some degree of moral authority although it too is subject to member states' alternative interpretations of their actions (or lack thereof). Countries that respond ineffectively to a global pandemic represent a threat to every other country.

### Pandemic Risk and Response

Covid was not the expected “big one:” a doomsday pandemogen that is highly contagious, spreads before symptoms, is dangerous and deadly, to which people have no immunity, for which no vaccines or treatments exist, and has an animal reservoir: a contemporaneous grim-reaper. Such a pandemogen poses an existential threat whether it emerges from a zoonotic disease, evolves from an existing human pathogen, or is manufactured in a laboratory. People may hope that the “big one” will not happen in their life-time, but hope is not a sensible strategy. A realistic PPB is needed; geared to an ever-evolving contextual background. In the future, as in the past, NPI will remain the first defense against the spread of pandemogens. As societies become more complicated and interconnected, NPI may be increasingly difficult to implement and to sustain; the accompanying infodemic, more difficult to counter. Advances in digital-age technologies can be expected to facilitate PRM, but their use may raise troubling security, privacy, and related issues. When the next pandemic strikes preparedness among countries will likely again be uneven. Some governments may strengthen different aspects of pandemic preparedness and response capabilities, but fall short of a realistic PPB. Others may not start (due to their socio-political traditions or because they lack sufficient resources).

### Beginning Again

Articles, books, and reports about what went right and what went wrong, and why, started to roll off printing presses immediately after covid began. This telldemic is sure to continue for years to come. Doubtless, authors will apportion blame (and there is plenty of blame to spread around) and will make recommendations; many will be aspirational. Leaders, especially those who mismanaged covid, may try to deflect blame, saying the experts made me do it (and they got it wrong). There was a gap of 100 years between the 1918 pandemic and covid but global pandemics should not be thought of as 100-year events. The next pandemic might already be emerging. Unless the world can better manage climate change, land use and other environmental factors that bring people in closer contact with animals, the risk of pandemics can only increase. Unless mechanisms exist to transform recommendation into practical actions, the pandemic panic cycle will repeat itself: (1) react with surprise, then panic, (2) muddle through, (3) perceive the end, and (4) move-on (until the next one strikes). Once covid is considered to have ended, collective amnesia may set in as the pandemic recedes in the rear-view mirror, becoming a fading memory lost in the mists of history. Hopefully, when the next pandemic strikes, policy decision-makers will not be reading this article and lamenting “we should have done that”—all the more reason to begin a pandemic playbook when a pandemic is ending.

## Data Availability Statement

The original contributions presented in the study are included in the article/supplementary material, further inquiries can be directed to the corresponding author/s.

## Author Contributions

The author confirms being the sole contributor of this work and has approved it for publication.

## Conflict of Interest

The Author is the Founder/President of the World Development Group, Inc., a consultancy. In writing the article, the author was not influenced by any commercial or financial relationships that could be construed as a potential conflict of interest.

## Publisher's Note

All claims expressed in this article are solely those of the authors and do not necessarily represent those of their affiliated organizations, or those of the publisher, the editors and the reviewers. Any product that may be evaluated in this article, or claim that may be made by its manufacturer, is not guaranteed or endorsed by the publisher.
